# A rare cutaneous neoplasm in an elderly patient

**DOI:** 10.1016/j.jdcr.2024.08.038

**Published:** 2024-09-19

**Authors:** Nujood Alzahrani, Zachary Wolner, Douglas Parker, Travis W. Blalock

**Affiliations:** Department of Dermatology, Emory University School of Medicine, Atlanta, Georgia

**Keywords:** adenosquamous carcinoma, cutaneous

## Case description

**Fig 1:**
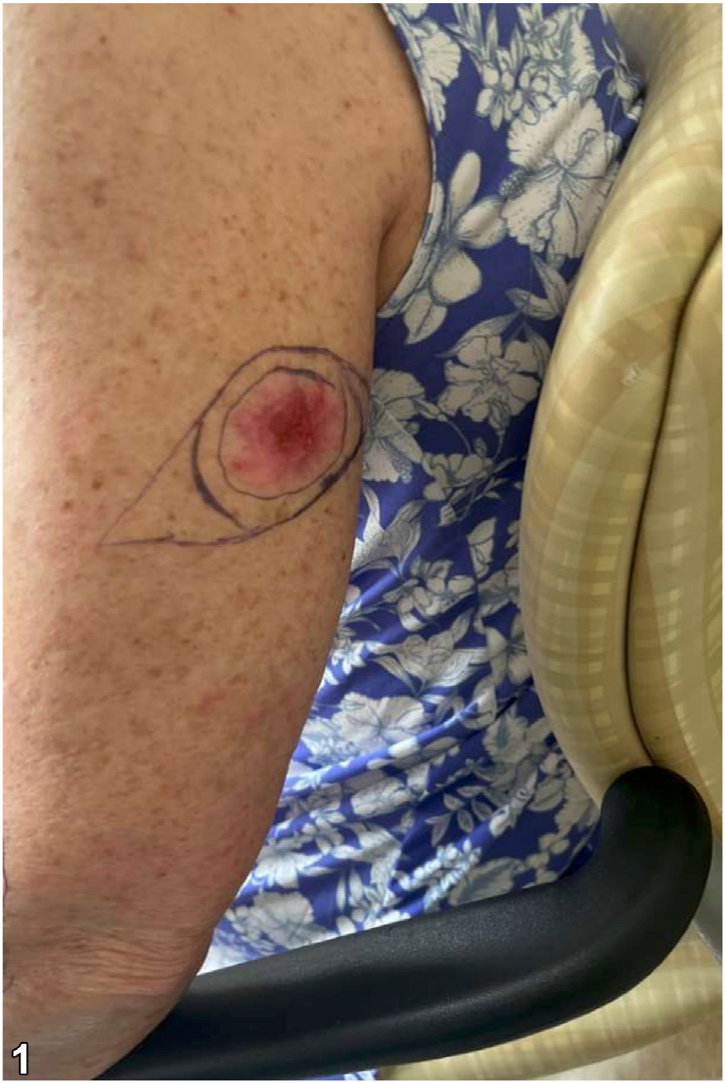

**Fig 2:**
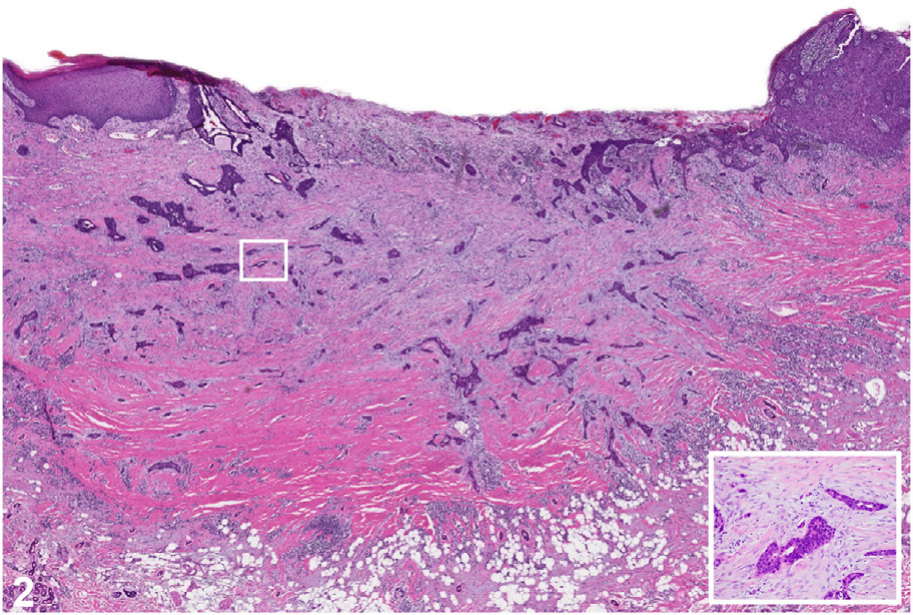

**Fig 3:**
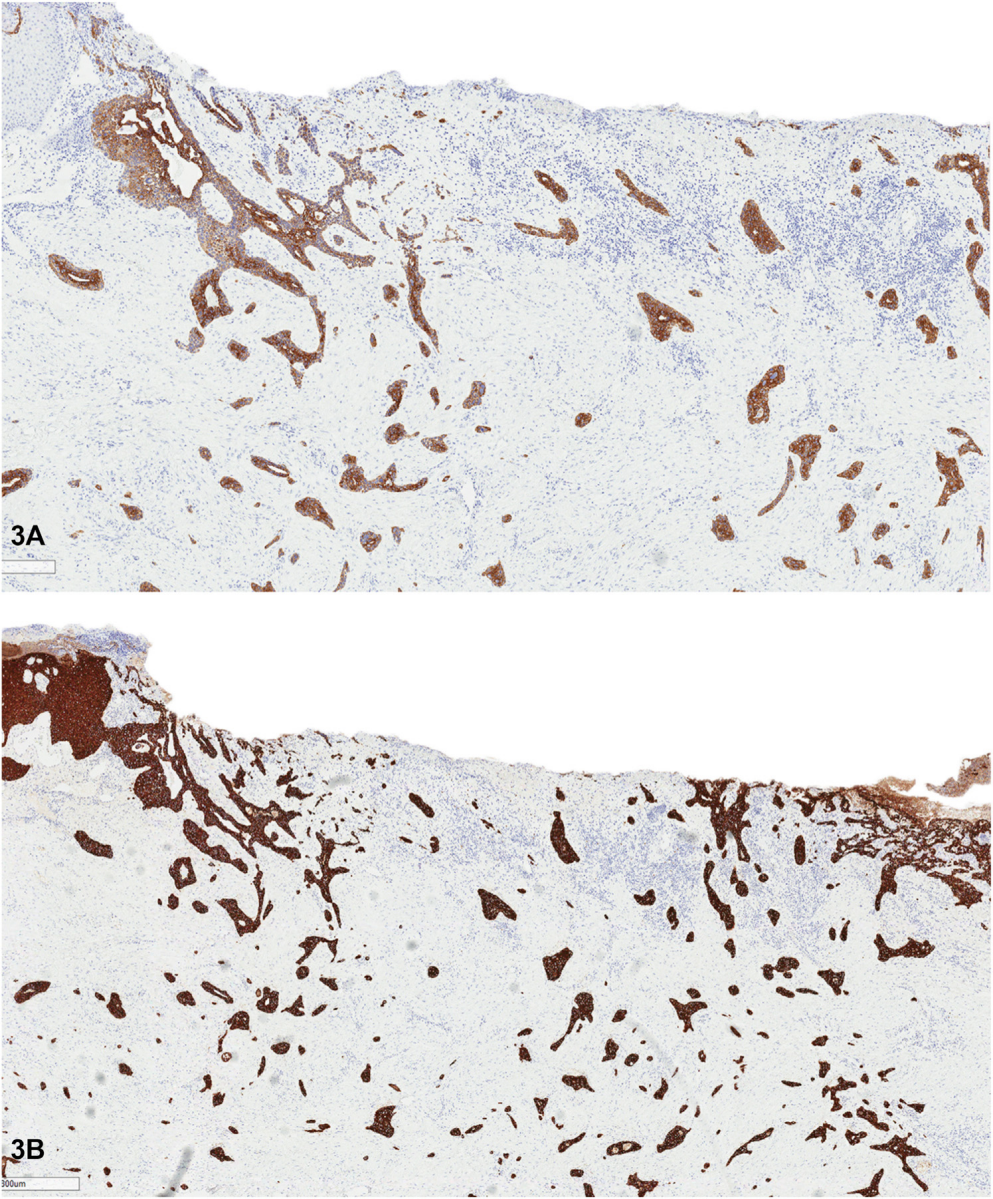

A 68-year-old patient with a history of multiple basal cell carcinomas on the trunk and extremities presented with an enlarging, tender, fleshy plaque on the left arm. The clinical lesion, histopathology, and immunohistochemical (IHC) images are shown below. [Fig 1, Fig 2, Fig 3].

**Question 1: What is the most likely diagnosis?**

A. Acantholytic squamous cell carcinoma.
B. Metastatic adenocarcinoma.
C. Adenosquamous carcinoma.
D. Desmoplastic squamous cell carcinoma.
E. Basal cell carcinoma with squamous differentiation.

**Answers:**

A. Acantholytic squamous cell carcinoma – Incorrect. Acantholytic squamous cell carcinoma is an epidermal-based proliferation with areas of acantholysis forming pseudo-glandular spaces.^1^
B. Metastatic adenocarcinoma – Incorrect. An epidermal connection is supportive of a primary cutaneous neoplasm. Cutaneous metastasis accounts for 2% of all skin neoplasms. The most common visceral malignancies to metastasize to the skin are breast cancer in women and lung cancer in men. Both tumors can exhibit glandular structures on pathology and commonly metastasize to the chest.^2^
C. Adenosquamous carcinoma – Correct. Adenosquamous carcinoma is a rare high-grade variant of squamous cell carcinoma. It predominantly affects men, with an average age of 74 years. This malignancy typically arises in sun-exposed areas, primarily involving the head and less commonly the extremities. Histologically, these tumors are characterized by an infiltrative pattern of irregular islands of squamous cells, often originating in the epidermis, with features of keratinization and areas of ductal and glandular differentiation.^1^^,^^3^
D. Desmoplastic squamous cell carcinoma – Incorrect. Desmoplastic squamous cell carcinoma is a rare, aggressive type of squamous cell carcinoma. Histopathological findings include epithelial cells forming cords and trabeculae that infiltrate a dense desmoplastic stroma. The presence of keratin pearls and perineural involvement is also observed.^4^
E. Basal cell carcinoma with squamous differentiation – Incorrect. Basosquamous carcinoma, a controversial entity, is a cutaneous malignancy with features of a squamous cell carcinoma and a basal cell carcinoma. These neoplasms display 2 populations of blue basaloid islands and eosinophilic squamous cells with a transitional zone in between.^5^

**Question 2: What staining pattern is expected in this entity?**

A. Positive for cytokeratin 7 (CK7), cytokeratin 5/6, carcinoembryonic antigen (CEA), and negative for cytokeratin 20.
B. Positive for Ber-EP4 and epithelial membrane antigen.
C. Positive for CK7, progesterone receptor, and estrogen receptor, and negative for cytokeratin 5/6.
D. Positive for cytokeratin 5/6 and negative for CK7 and CEA.
E. Positive for CK7, carcinoembryonic, thyroid transcription factor-1, and negative for cytokeratin 5/6.

**Answers:**

A. Positive for cytokeratin 7 (CK7), cytokeratin 5/6, carcinoembryonic antigen (CEA), and negative for cytokeratin 20 – Correct. Adenosquamous carcinoma demonstrates 2 distinct staining patterns. The squamous component is highlighted by cytokeratin 5/6 (CK5/6). This transitions into the adenosquaomus component, which is positive for CK7 and CK5/6. CEA can be used to highlight ductal differentiation. Immunohistochemistry for cytokeratin 20 is negative, excluding certain metastatic adenocarcinomas.^1^^,^^3^
B. Positive for Ber-EP4 and epithelial membrane antigen – Incorrect. Basosquamous carcinomas can be identified through IHC staining, which typically reveals a combination of Ber-EP4, commonly found in basaloid component, and positive epithelial membrane antigen, typical of squamous component.^5^
C. Positive for CK7, progesterone receptor, and estrogen receptor, and negative for cytokeratin 5/6 – Incorrect. IHC staining of metastatic breast adenocarcinoma often demonstrates positive staining for CK7, progesterone, and estrogen receptors, while negative for CK5/6. Additional staining and clinical history may be needed to fully distinguish metastatic breast carcinoma from a primary cutaneous adnexal carcinoma.^2^
D. Positive for cytokeratin 5/6 and negative for CK7 and CEA – Incorrect. Acanthylotic squamous cell carcinomas lack true glandular differentiation; hence, they are less likely to exhibit positive staining for CK7 or CEA.^1^
E. Positive for CK7, carcinoembryonic, thyroid transcription factor-1, and negative for cytokeratin 5/6 – Incorrect. Metastatic lung adenocarcinoma would exhibit the following staining pattern: positive for CK7, CEA, and thyroid transcription factor-1, and typically negative for CK5/6.^2^

**Question 3: What of the following is true regarding this diagnosis?**

A. Metastasis is common.
B. Diagnosis of this tumor indicates an overall poor general prognosis.
C. Tumor is locally aggressive with high recurrence risk.
D. Immunosuppression is unlikely to impact recurrence risk.
E. Radiation is the best initial treatment for a localized lesion.

**Answers:**

A. Metastasis is common – Incorrect. Distant metastasis in adenosquamous carcinoma is an exceedingly rare occurrence. Reported sites of metastasis have included lymph nodes and soft tissues in close proximity to the primary tumor.^1^^,^^3^
B. Diagnosis of this tumor indicates an overall poor general prognosis – Incorrect. Adenosquamous carcinomas have a propensity for local recurrence and very rare distant metastasis.^1^^,^^3^ The presence of metastasis is indicative of a poorer prognosis.
C. Tumor is locally aggressive with high recurrence risk – Correct. Adenosquamous carcinomas are associated with a risk of recurrence, necessitating close monitoring of affected patients. Factors that suggest an increased risk of recurrence include deep invasion, microscopic peri-neural invasion, and immunosuppression.^3^
D. Immunosuppression is unlikely to impact recurrence risk – Incorrect. Immunocompromised individuals have been found to be at an elevated risk for recurrence of adenosquamous carcinoma.^3^
E. Radiation is the best initial treatment for a localized lesion – Incorrect. The primary approach for adenosquamous carcinoma is surgical intervention with local excision or Mohs micrographic surgery. However, due to the rarity of the tumor, no standard treatment exists. Radiotherapy and epidermal growth factor receptor antagonists (eg, cetuximab) can be employed as adjunctive treatments for locally advanced cases.^3^

## Conflicts of interest

None disclosed.
